# Evaluation of the Sensory and Textural Properties of Cheese-Containing Frankfurters Available on the Polish Market

**DOI:** 10.3390/foods15020226

**Published:** 2026-01-08

**Authors:** Kacper Kozłowski, Michał Piątek, Mirosława Krzywdzińska-Bartkowiak, Agnieszka Bilska

**Affiliations:** Department of Meat Technology, Faculty of Food Science and Nutrition, Poznan University of Life Sciences, Wojska Polskiego 31, 60-624 Poznan, Poland; michal.piatek@up.poznan.pl (M.P.); miroslawa.krzywdzinska-bartkowiak@up.poznan.pl (M.K.-B.); agnieszka.bilska@up.poznan.pl (A.B.)

**Keywords:** frankfurters, cheese, sensory properties, texture, meat products

## Abstract

The increasing trend in the consumption of milk and dairy products, as well as meat and meat-derived products, may be one of the factors contributing to the growing popularity of cheese-added frankfurters, which combine these two product categories. The aim of this study was to compare the textural, colour, and sensory properties of commercially available cheese-containing frankfurters using instrumental measurements and descriptive sensory analysis. The study focused on ready-to-eat products with naturally varying formulations to identify measurable differences in quality attributes and explore potential associations between composition and product characteristics. Instrumental methods were applied, including texture profile analysis (TPA), the Warner–Bratzler shear force test (WBSF), and CIE L*a*b* colour measurement, along with quantitative descriptive analysis. The results confirmed significant differences between the samples in terms of texture and flavour. Notably, Sample B showed the highest shear force (2.91 N), while Sample C exhibited the lowest (1.82 N). Samples A and D, both containing 12% processed cheddar cheese, had the highest b* values (30.1 and 22.4, respectively), which corresponded to their more intense yellow colour and higher scores for cheese flavour. The addition of cheese had a beneficial effect on product acceptability; however, the final outcome depended on the form and amount of cheese, as well as other ingredients. These findings suggest that cheese may serve as a valuable additive to homogenised meat products, enhancing sensory appeal without compromising technological quality. Future studies will compare different cheese types and concentrations and include consumer testing on a larger sample.

## 1. Introduction

The contemporary food market is characterised by considerable dynamics and diversity in the products on offer. One may easily find a single type of product on shop shelves in various flavours, weights, or even in interesting limited edition packaging. Adapting the products offered by food manufacturers in times of advancing globalisation and changing consumer attitudes poses a significant challenge. The consumer profile is changing as a result of the improvement of Poland’s economic situation. Therefore, the average food purchaser is better educated, wealthier and, thus, more demanding and aware of their food choices. For these reasons, consumer goods companies are taking steps to make their products more attractive, without necessarily creating completely new, innovative versions. It is observed that well-known and popular products are being given new characteristics, e.g., in terms of their flavour [1,2,3,4].

Global economic development affects the wealth of citizens, especially in countries where population growth is high, contributing to an ever-increasing share of meat and meat-derived products in the structure of related foods [5]. This leads to an expansion of the range of meat-derived products on offer and a constant search by their producers for a market niche that may be filled. An unquestionable challenge in this area is the creation, improvement and promotion of safe meat-derived products characterised by high quality, but also attracting consumers with their creativity and attractive sensory qualities. Consumers in the meat industry are most likely to choose fresh meat (49%), followed by sausage products (38%) [6]. When observing the market, one can see that producers are making the aforementioned efforts to respond to the continuously growing demands of consumers. There are at least several types of frankfurters: protein frankfurters and also ones with reduced fat content, which are targeted at individuals who practice sports and look for sources of protein, while at the same time being careful about the calorie content of the product. Frankfurters for children—often with colourful graphics and slogans popular among this age group, frequently characterised with lower weight or duo-pack packaging. “Pure” or “Natural” frankfurters are designed with the concept of the so-called ‘clean label’ in mind. They are usually marked with additional information about the absence of preservatives (including sodium nitrate) or the percentage of meat used in production. The most interesting examples of frankfurter product diversification are those with various flavour additives—a widely available group is frankfurters with added cheese [7,8,9].

Although several studies have examined the use of cheese powders in processed meats, little is known about the quality attributes of commercial frankfurters that contain actual cheese pieces. These products vary considerably in formulation, including differences in meat content, cheese type, and the use of functional additives. However, no comprehensive comparative studies have evaluated how this compositional diversity affects their texture, colour, or sensory characteristics. Unlike previous research based on controlled laboratory formulations, the present study investigates ready-to-eat commercial products, offering practical insights into their sensory appeal and technological properties. To the best of our knowledge, no previous study has comprehensively examined the combined influence of cheese type and form on the sensory and textural characteristics of ready-to-eat frankfurters. This study, therefore, fills an important research gap by providing comparative data from commercial products. This approach is particularly relevant for researchers and food industry professionals aiming to improve product quality and consumer satisfaction. By combining instrumental texture and colour analysis with descriptive sensory evaluation, this study addresses a clear gap in the current literature. This additive may be an alternative to the traditional flavour enhancers and functional agents used in meat processing. The use of cheese powder in the production of cold meats creates new opportunities for shaping sensory characteristics, while reducing the sodium content in the finished product. Cheese powder is produced using a cheese emulsion, which is obtained by dissolving cheese in water with the addition of calcium sequestrants, such as sodium citrate or phosphates. The emulsion is then homogenised, and the resulting mixture is dried at a high temperature. The obtained power should have a homogenous structure, good solubility, oxidative stability and an intense characteristic flavour profile. Cheese powder is convenient to store, has a long shelf life and may be easily incorporated into recipes both in a dry form and after hydration. Therefore, it is used not only in the cheese industry, but also in the meat processing, baking and catering sectors [10,11,12].

The results of other studies on cheese powder have shown that frankfurters with the addition of selected cheese powders—especially mixtures of hard cheese and blue cheese—had a significantly higher saltiness rating compared to the control sample, even though the salt content in all variants was very similar. This suggests a potential enhancing effect that could be utilised in the production of reduced-sodium meat-derived products. Interestingly, the evaluators did not report a noticeable cheese flavour in the products, despite the fact that the cheese powder came from flavour-intensive types of cheese. This indicates that cheese powder may act as a natural flavour enhancer without affecting the characteristic sensory profile of the product. Furthermore, the addition of cheese powders was observed to influence the texture of frankfurters, particularly in terms of increasing the sensation of hardness. Although no instrumental analysis was performed, the authors suggest the possibility of cross-interaction between the perception of taste and texture [13].

The consumption of milk and dairy products, including cheese, has been on the rise in recent years. According to data contained in the 2024 report of the Central Statistical Office, the production of cheese and curd cheese in 2023 increased by 35,000 t compared to the previous year and amounted to 853,000 t—this figure is the result of the amount of cheese and curd cheese produced in Poland reduced by exports and increased by imports. On the other hand, the consumption of milk and dairy products per capita was 267 L/person, which represents an increase in consumption of 5.07% compared to 2022 [14]. The upward trend in the consumption of milk and dairy products, as well as meat and meat-derived products, may be one of the reasons for the popularity of frankfurters with cheese, as they combine the two product groups mentioned above. It may therefore be hypothesised that the addition of cheese to frankfurters available on the market significantly affects their textural properties and sensory profile, improving consumer acceptance.

Taking into account the above trends, the aim of this study was to compare the textural, colour, and sensory properties of commercially available cheese-containing frankfurters using instrumental measurements and descriptive sensory analysis. The study focused on ready-to-eat products with naturally varying formulations to identify measurable differences in quality attributes and explore potential associations between composition and product characteristics.

## 2. Materials and Methods

### 2.1. Material

The research material consisted of cheese-containing frankfurters available on the Polish market, purchased in local supermarkets. Five different brands of frankfurters were coded with letters A–E. All products were stored in refrigerated conditions, in accordance with the conditions recommended by the manufacturer. Individual packages of each brand came from the same production batch. For each product variant (brand), ten replicate measurements were performed for each instrumental parameter. Each measurement was conducted on a separate frankfurter from the same production batch to ensure representativeness. The analysed parameters included Texture Profile Analysis (TPA), Warner–Bratzler shear force (WBSF), and colour measurements. All analyses were carried out following the procedures described below.

As the tested products were commercially available and differed in formulation and unknown processing parameters, the observed differences in sensory and instrumental results cannot be attributed solely to cheese content. Other factors, such as starch, phosphates, fat content, or manufacturing processes, may have contributed to the measured values.

Cheese types are presented as described on the product labels (Table 1). Both the terminology and the type of cheese may vary across brands (e.g., “processed cheddar cheese,” “ripened cheese,” “hard cheese”). Quantitative cheese content is provided where available. Based on the order of ingredients, the cheese content in Brand B can be estimated to exceed 2%, likely falling within the 3–6% range, although the exact percentage is not declared. This estimation remains approximate and should be interpreted with caution.

### 2.2. Methods

**Warner–Bratzler shear force test (WBSF)**

The shear force was measured using a TA.XT.plus texture analyser (Stable Micro Systems, Godalming, United Kingdom) with a Warner–Bratzler cutter. Samples with a diameter of 1.27 × 10^−2^ m and a height of 4 × 10^−2^ m were tested. The head travel speed was 0.5 m/min.

The maximum shear force [N] and shear work (area under the curve, [N·s]) were read from the recorded force-time curve.

The procedure was performed in accordance with AMSA recommendations [15].

**Texture Profile Analysis (TPA)**

The texture profile analysis of cheese-containing frankfurters was performed using the double compression method [16]. In accordance with the principles described in PN-EN ISO 11036:2020-08 [17]. The measurement was performed using a TA.XT.plus texture analyser (Stable Micro Systems, Godalming, United Kingdom) equipped with a P/10 attachment and an HDP/90 specimen table. Samples with a diameter of 1.27 × 10^−2^ m and a height of 1.0 × 10^−2^ m were subjected to double compression to 50% of their original height at a head speed of 0.5 m/min.

The texture parameters were determined from the force-deformation curve: hardness 1 and 2 [N], springiness, gumminess, chewiness, resilience, adhesiveness [N·s], cohesiveness.

CIE L*a*b* colour measurement

The colour of the cross-section of the cheese-added frankfurters was determined using a SpectroPen portable reflectance spectrophotometer (Dr. Lange, Düsseldorf, Germany). The measurements were conducted in reflection mode, using d/8° geometry (integrating sphere), a D65 illuminant and a standard 10° observer, in accordance with ISO/CIE 11664-4:2019 [18].

The instrument was calibrated before each series of measurements using a white standard provided by the manufacturer.

Ten independent measurements were taken on each sample at randomly selected points on the surface. The average values of the CIE L*a*b* colour coordinates were then calculated.

The colour differences (ΔE) between the samples were determined.

**Quantitative Descriptive Analysis (QDA)**

Sensory tests were conducted in a sensory analysis laboratory equipped with individual booths (at a controlled temperature of 21 ± 1 °C and combined natural/artificial light) designed in accordance with ISO 8589:2009 [19]. The laboratory was located at the Department of Gastronomy and Functional Food Technology of the University of Life Sciences in Poznań (Poland). Sensory characteristics were assessed by a 9-person trained panel ISO 6564:1985 [20] from the Faculty of Food Sciences and Nutrition, using quantitative descriptive analysis. A total of 20 descriptors were evaluated, including basic characteristics of colour (intensity, uniformity), texture (hardness, lumpiness, binding, juiciness), taste (fat flavour, meat flavour, salty flavour, off-taste, seasoning flavour, smoky flavour, cheesy flavour), aroma (fat aroma, meat aroma, smoky aroma, seasoning aroma, off-aroma, cheesy aroma) and an overall assessment. Descriptors of colour, texture, taste and aroma were selected in preliminary tests. The study used an unstructured linear scale with marked values ranging from 0 to 10 conventional units (c.u.), where 0 indicated no intensity of a given characteristic, and 10 indicated high intensity of a given characteristic. The product samples were coded with random three-digit numbers and served on odourless white plates. Water was used to rinse the mouth between samples. All samples were evaluated in two independent repetitions. Participants were informed about the purpose of the study and that their participation was entirely voluntary, meaning that they could discontinue the analysis at any time and that their responses would be anonymous. The panel size was selected in accordance with published recommendations for Quantitative Descriptive Analysis (QDA), which typically suggest 8–12 trained assessors to ensure reliability and discriminative ability. Prior to the study, panellists participated in refresher training sessions and calibration using reference samples to align interpretations of each descriptor. The Ethics Committee of the Poznań University of Life Sciences issued ethical approval for human participation in this study (Approval No. 4/2025, granted on 20 March 2025).

**Statistical analysis**

The numerical data obtained in the research were subjected to statistical analysis using Statistica software (Data Analysis Software System, StatSoft, version 13.3). The normality of the data distribution was verified using the Shapiro–Wilk test, and the homogeneity of variance was verified using Levene’s test. For data meeting the assumptions of normality and homogeneity of variance, one-way analysis of variance (ANOVA) was used. If significant differences between groups were found, Tukey’s post hoc test was performed to determine between which samples there were statistically significant differences. For non-parametric results, the Kruskal–Wallis test, which is the non-parametric equivalent of ANOVA, was used. After demonstrating the significance of the differences, multiple comparisons of mean ranks were performed to indicate the differences between individual groups.

Only the Warner–Bratzler shear force (WBSF) data met the assumptions of parametric testing and were therefore analysed using one-way ANOVA, with Tukey’s post hoc test applied when significant differences were found. These results are reported as Mean ± SD.

All other data did not meet parametric assumptions and were analysed using the Kruskal–Wallis test, followed by multiple comparisons of mean ranks. For these variables, the results are presented as medians with interquartile ranges.

To deepen the interpretation of the results of the QDA evaluation, boxplots were prepared to show the variability for statistically significant parameters. For data that did not meet the assumptions of normal distribution, the results were presented as the median and interquartile range (IQR) instead of the mean and standard deviation. This approach, recommended in the literature, minimises the impact of outliers and ensures greater reliability in the interpretation of results [21].

## 3. Results and Discussion

### 3.1. Assessment of Textural Properties—WSBF

Table 2 presents the average shear force values for different brands of cheese-containing frankfurters. The Warner–Bratzler shear test was used to assess the texture of cheese-containing frankfurters. This method allows for accurate recording of the force required to cut a sample, which reflects characteristics such as hardness, brittleness and cohesiveness of the product [22].

The greater the shear force and work, the greater the sensation of hardness and resistance of the frankfurters during consumption. The highest shear force was recorded for brand B frankfurters (2.91 N), which indicates the highest hardness among the frankfurters tested. This value differed statistically significantly from frankfurters of brand C (1.82 N), which had the lowest shear force and thus the lowest hardness. Frankfurters of brands A, D and E achieved intermediate values, with samples of brands A and D not differing significantly from each other or from brand B, while brand E differed significantly from C, but not from A or D. Similar correlations are observed for the shear work parameter.

In a study conducted by Piotrowska et al. (2005), where the Warner–Bratzler shear force was measured on frankfurters produced in a processing plant with the following composition: 50% class III pork, 21.4% fine fat, and 28.6% water, a shear force value of 4.447 N was obtained for the control sample [23]. The researchers used an Instron 1140 texture analyser.

Although this value was higher than those recorded for the cheese-containing frankfurters in the present study, such differences should be interpreted with caution. They may stem from multiple factors, including formulation, additive use, processing conditions, and measurement methodology. While higher meat content could theoretically lead to increased shear force, this relationship is not always linear in processed meat products due to the complex interaction of ingredients and production variables.

In samples A and D, despite having nearly 34 percentage points more meat than the control frankfurters from Piotrowska et al., the shear force values were still lower by approximately 1.5 N. This may reflect the combined influence of cheese content, texturising agents, and other formulation differences, though no single factor can be isolated as the cause.

Several compositional and technological factors, beyond meat content alone, may influence the texture of frankfurters. These include water content and functional additives such as starches or phosphates (Table 1). Florek et al. (2015) [24] also demonstrated that replacing part of the fat with potato starch can reduce shear force by modifying water binding and structural density.

In a study by Matulis et al. (1994), an average shear force of 2.18 kgf (21.39 N) was reported for cold frankfurter samples from a single brand available on the market. However, this value cannot be directly compared with those in the present study due to differences in sample formulation, testing temperature, and the use of an Instron 1122 device. Only water (51.89%) and fat content (29.97%) were disclosed [25].

Similarly, Domaradzki et al. (2012) reported shear force values of 26.03 N and 24.6 N for frankfurters with 93% and 71% pork content, respectively, using a Zwick/Roell ProLine Z0.5 testing machine [26]. These results fall above the values observed in the present study.

Collectively, these external data illustrate the considerable variability in shear force values across commercial meat products. However, differences in formulations, measurement instruments, and sample handling limit the reliability of direct comparisons. Notably, in the current study, higher meat content did not always correspond with greater shear force. For example, sample B, containing 72% pork and hard cheese, showed the highest WBSF value among the tested products. In contrast, sample E, with the lowest meat and one of the highest cheese contents, exhibited the lowest shear force. These observations may reflect the combined effect of other functional additives, such as sodium alginate, which is known to form firm gels and influence textural outcomes. Still, due to the complex nature of product formulations, no single factor can be isolated as solely responsible for the observed differences.

Based on the declared compositions of the tested frankfurters (Table 1), several functional additives commonly used in meat processing may contribute to the observed differences in texture. Among these, phosphates—such as triphosphates and diphosphates—were found in products A, B, D, and E. According to literature, phosphates are known to stabilise muscle proteins, enhance water retention, and improve the cohesiveness of meat matrices [27]. Their presence might be expected to increase texture parameters such as shear force. In product B, which contained diphosphates and exhibited the highest WBSF value (2.91 N), this effect may be reflected. However, in samples A (2.74 N) and D (2.63 N), which contained triphosphates, the shear force was slightly lower, suggesting that the relationship is not consistent across products and may be modulated by other formulation components.

Collagen and pork proteins were also listed as ingredients in products A, C, and D. These proteins increase the total protein content and, upon thermal processing, could influence product structure and firmness. Collagen, due to its low solubility, is often associated with a firmer, more compact texture. Nevertheless, product C—which contained collagen proteins—had the lowest measured shear force (1.82 N). This discrepancy likely reflects the influence of multiple formulation or processing factors, and not collagen alone.

Another additive frequently used in production is starch, which is present in all variants of the frankfurters tested. It acts as a filler and stabiliser, and also binds water. The addition of starch affects the softness and resilience of frankfurters, limiting the leakage of juices during thermal processing. Dolata et al. (2000) showed that replacing part of the fat with modified potato starch resulted in a significant reduction in shear force, which was the effect of increased hydration and lower density of the structure [28].

### 3.2. Assessment of Textural Properties—Texture Profile Analysis (TPA)

The TPA method is widely used in the testing of meat-derived products, including cold meats, as it enables precise assessment of texture properties across a wide range of tested parameters. Like WBSF, TPA correlates with sensory assessments of texture and facilitates quality control at the production stage [29].

Parameters such as adhesiveness [N·s], springiness, cohesiveness, gumminess, chewiness, resilience, and hardness 1 and 2 [N] were assessed.

According to Table 3, the most notable differences between the samples were observed in terms of adhesiveness, chewiness, and gumminess. Frankfurters from brand A showed the lowest adhesiveness value (−0.379), indicating a clear structural breakdown during deformation, which was also reflected in their lower resilience and cohesiveness. Together with brand D, these frankfurters contained the highest amount of processed cheddar cheese (12%), which may have contributed to a softer, creamier sensation.

As noted by Guinee (2016), the incorporation of processed cheese into meat matrices may lead to texture softening due to the release of fat and moisture during cooking. This can interfere with the formation of the myofibrillar protein gel network, disrupting protein–protein interactions and resulting in lower mechanical resistance and a creamier mouthfeel. These mechanisms may explain the lower hardness and adhesiveness values observed in high-cheese samples, despite their relatively high meat content [30].

In contrast, brand B samples exhibited the highest hardness (21.08 N), gumminess, and chewiness, which may be associated with a denser protein matrix and potentially lower cheese content. In such formulations, the absence or limited amount of cheese may allow for more extensive protein cross-linking, resulting in a firmer texture. Moreover, partially melted cheese or aged cheese fragments—as found in brand E—can also act as a reinforcing element, especially if not fully integrated into the meat emulsion. Brand E, despite having the lowest total meat content (52%) and a blend of poultry meat and ripened cheese, displayed the highest values for springiness, gumminess, and chewiness [30].

These findings reinforce that the relationship between cheese addition and texture is not linear but highly dependent on cheese type, structure, moisture and fat content, and its interaction with the surrounding protein matrix. Even samples with similar meat and cheese content (A and D) differed in textural parameters, highlighting the complexity of formulation-based effects and the need for cautious interpretation of the results.

Moreover, the role of cheese in influencing the flavour profile of frankfurters should not be limited to its intensity or quantity alone. In particular, ripened cheeses, such as the one used in sample E, are known to be rich in volatile compounds, including ketones, aldehydes, esters, and free fatty acids. These molecules contribute to sensory attributes such as umami, sharpness, or buttery notes and may interact synergistically with meat-derived compounds to enhance the perceived flavour complexity of the product. Although sample E showed relatively low perception of cheese-specific flavour and aroma, its high overall rating and meat flavour intensity may be partially explained by the presence of ripened cheese, which is known to carry a broad spectrum of flavour-active compounds that do not always translate to a strong “cheesy” note but can enhance savouriness and mouthfeel [31].

### 3.3. Assessment of Colour Properties in CIE L*a*b* Space

According to Table 4, the highest L* value (57.40) was characteristic of brand C frankfurters, which indicates a lighter, more uniform colour of the product. Brands B (L* = 53.75) and E (L* = 52.00) also achieved relatively high brightness values.

Whereas brand A had by far the lowest L* value (41.25) and differed statistically significantly from all frankfurters except brand D, suggesting a darker, more intense colour. This may be due to the addition of smoke flavouring and a higher proportion of red colour (a = 9.55*), associated with a higher proportion of pork. The a* parameter, which indicates the saturation of red colour, also reached its highest value in products of brand A (9.55) and brand D (8.35). These values were significantly higher than in samples of brand C (4.40) and brand B (5.30), which were characterised by a lighter but less saturated shade of red. The intensity of the red colour in meat-derived products depends mainly on the presence of myoglobin, which is a typical pigment found in meat. In the case of brands A and D, which had the highest meat content in the ingredients composition declared by the manufacturers, it is reasonable to conclude that the redder colour was a result of the highest pork content among the frankfurters tested.

The b* parameter value was highest in frankfurters of brands A (30.10) and D (22.35), which is directly related to the addition of processed cheddar cheese (12%), which has a yellow colour. Products of brands C, B and E, containing other types of cheese (La Maar, hard, ripened), showed lower b* values, which confirms the influence of the type of cheese on the colour of the finished product. In order to more accurately determine the colour differences between the frankfurters tested, the ΔE parameter was calculated. The ΔE index allows the colour difference between samples to be assessed on the basis of CIE L*a*b* data in accordance with Formula (1) [32].

Formula for calculating the total colour difference (ΔE) in the CIE L*a*b* colour space.

(1) $$\Delta E=\sqrt{(\Delta L^{*}{)}^{2}+(\Delta a^{*}{)}^{2}+(\Delta b^{*}{)}^{2}}$$

Depending on the context in which ΔE comparisons are applied, interpretation thresholds may vary. Commonly cited guidelines include the following:

ΔE < 1: Difference not perceptible to the human eye;
1 ≤ ΔE < 2: Difference perceptible only to a trained observer;
2 ≤ ΔE < 3.5: Difference perceptible to most observers;
3.5 ≤ ΔE < 5: Clear visual difference;
ΔE ≥ 5: Colours are perceived as distinctly different.

**Figure 1:**
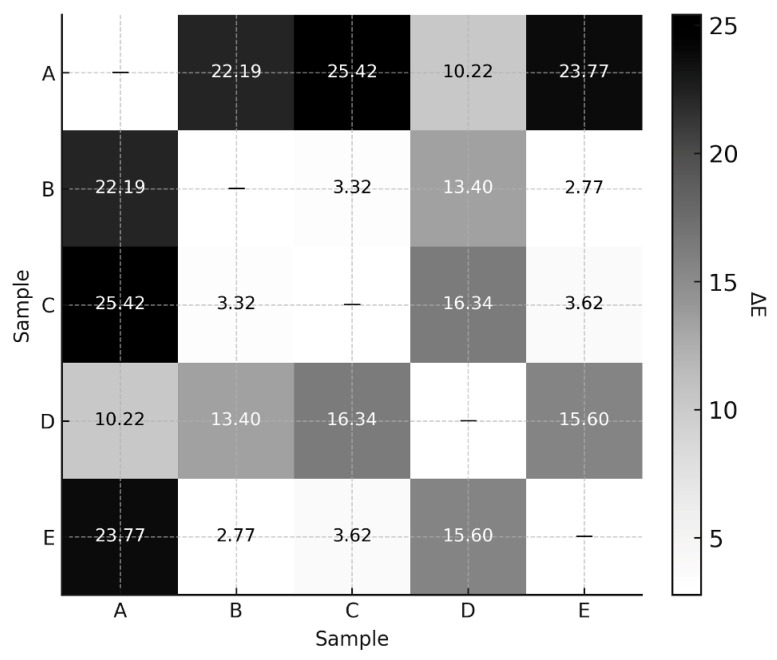
ΔE parameter difference matrix for cheese-added frankfurters. A–E represent the codes of the brands of cheese-added frankfurters.

The ΔE value reflects the Euclidean distance between two points in the CIE L*a*b* space and is commonly used to quantify colour differences. The ΔE matrix analysis (Figure 1) shows that the largest instrumental colour differences occurred between brands C (ΔE = 25.76), E (ΔE = 23.14), and B (ΔE = 21.84). These values indicate measurable differences in colour coordinates, which may result from compositional variations, such as processed cheddar cheese addition or meat content.

The smallest ΔE values were noted between brands B and E (ΔE = 3.13) and B and C (ΔE = 4.26). While these values fall within ranges often associated with perceptible differences, it is important to note that no consumer or visual evaluation was conducted in this study. Therefore, no assumptions are made about whether these differences would be noticeable or meaningful to consumers. The instrumental data are presented as indicators of colour variability only.

This result aligns with the relatively high L* values (above 52) and lower red and yellow saturation observed for these brands, in contrast to brands D and A. Domaradzki et al. (2012) reported values of L* = 67.77, a* = 12.48, and b* = 9.22 for frankfurters with 93% pork content, which are notably different from those of brand A in the present study (L* = 30.1, a* = 9.55, b* = 30.1), despite a similar meat content. The use of processed cheese, such as cheddar, may contribute to this divergence, particularly the elevated b* value [26].

Similarly, frankfurters with 71% pork content in the Domaradzki study (L* = 65.27, a* = 12.77, b* = 11.54) had higher lightness and lower yellowness than brand B in the current study (L* = 53.75, a* = 5.3, b* = 12.7). While these contrasts may suggest a potential influence of cheese addition, the differences should be interpreted cautiously due to variability in formulation, additives, and analytical conditions across studies. Therefore, the observed differences in colour parameters may reflect a combination of factors rather than the isolated effect of cheese [26].

### 3.4. Assessment of Sensory Properties—Quantitative Descriptive Analysis

Table 5 presents the median values ± interquartile range of the attributes of the quantitative descriptive analysis.

In terms of hardness, the product of brand B achieved the highest result (5.2), which is consistent with the instrumental results, where this product also distinguished itself with the highest values of hardness 1 and 2. High hardness may indicate a more compact structure and lower content of fat or other technological additives, such as the aforementioned starch. In contrast, brand C showed the lowest hardness (3.4), which is also reflected in the lowest shear force and TPA values, confirming the consistency of sensory data with instrumental measurements.

The sensory evaluation highlighted brand E as a particularly flavourful product, achieving high ratings in meat and seasoning aroma, which may be attributed to its diverse meat composition and the presence of ripened cheese. Its recipe also included flavour enhancers and multiple fat sources, which likely contributed to the favourable perception. Notably, this brand received the highest overall score, indicating strong consumer appeal within the panel.

While some sensory attributes showed noticeable variation across products—such as smoky flavour and aroma intensity (more pronounced in brands B and C)—others, like colour uniformity, were rated similarly across all samples. The low off-aroma scores in all samples indicate high raw material quality and consistent processing.

Interestingly, brand A showed the highest graininess score, despite also exhibiting low instrumental adhesiveness, which would typically suggest a smoother texture. Visual observation of the frankfurters’ cross-section, revealing dense and large cheese inclusions, may have influenced this perception.

Among the attributes with statistically significant differences were colour intensity, cheese flavour and aroma, and overall assessment. Brands A and D, which contained the highest levels of cheddar cheese, stood out in perceived cheese flavour, whereas brands with lower cheese content scored notably lower in this category.

Despite having more intense cheese flavour, brands A and D did not achieve the highest overall scores—this distinction went to brands E and B, possibly due to their stronger meat flavour or firmer texture. These findings suggest that while cheese contributes positively to flavour complexity, other sensory elements such as hardness or seasoning may ultimately drive consumer preference.

Statistically significant differences were also identified in terms of cheese aroma. The highest scores were recorded for samples A (7.5) and D (6.2), confirming the distinct presence of cheese notes in these products. In comparison, samples C (1.5) and E (2.5) received the lowest scores, even though the latter contained ripened cheese, which usually delivers a characteristic aroma. It may be assumed that in this case, stronger seasoning and smoking notes dominated the cheese aroma, limiting its perception.

Sample B (3.9) achieved a moderate cheese aroma value, which is consistent with its average flavour profile as shown in the full analysis. The characteristics discussed earlier, such as high hardness and moderate meat flavour, may suggest that this product is less attractive to consumers who prefer distinct cheese notes.

Samples A and D were clearly distinguished by the sensory panel in terms of colour, flavour and cheese aroma. In products with a lower or undefined cheese content, such as C, B and E, the perception of cheese characteristics was significantly lower. This confirms that both the type and amount of cheese, as well as interactions with other ingredients in the recipe (meat, seasonings, technological additives), affect the distinctiveness of the cheese profile in this type of product.

**Figure 2:**
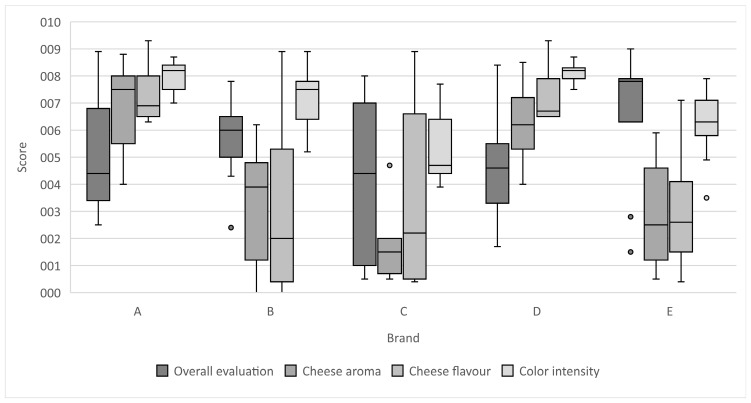
Comparison of statistically significant characteristics in quantitative descriptive analysis. A–E represent the codes of the brands of cheese-added frankfurters.

Figure 2 illustrates the distribution of sensory ratings using boxplots, which effectively present central tendency and variability for each sample. As Montgomery and Field emphasise, boxplots are an effective tool for comparing results in research groups, especially in the context of non-parametric analysis, where data may not meet the assumptions of normal distribution [31,32]. Furthermore, Montgomery highlights the importance of using this type of data comparison, especially where the data has a significant spread of values, and Field points out in his work their usefulness in survey research or methods involving subjective assessment, such as QDA [33,34]. Samples A and D stood out in attributes directly related to cheese addition, such as colour intensity, cheese flavour, and aroma, with high medians and narrow interquartile ranges, indicating consistent positive evaluation among panellists.

In contrast, sample E showed lower scores in cheese-related attributes but achieved the highest median in overall acceptability. This suggests that factors beyond cheese content—such as seasoning blend, meat type, or additive synergy—may have played a key role in overall preference. Sample C consistently exhibited low medians and high variability, suggesting a less favourable and more polarised reception.

**Figure 3:**
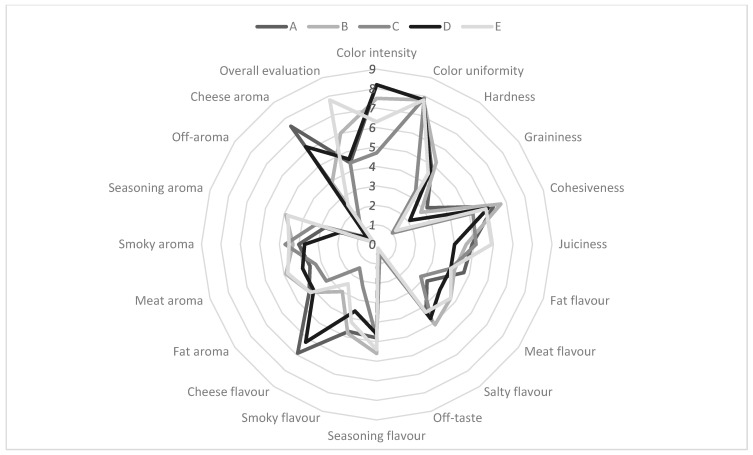
Comparison of sensory profiles of cheese-added frankfurters. A–E represent the codes of the brands of cheese-added frankfurters. The numbers represent the scale of intensity for each trait.

The radar chart (Figure 3) provides a comparative visual summary of sensory descriptors, highlighting the strengths of each product. This type of representation facilitates quick assessment of profile balance, in line with approaches used in prior studies on meat product evaluation [35].

Figure 3 shows that sample E has one of the most complex sensory profiles, particularly in terms of seasoning flavour intensity, smoky aroma, juiciness and cohesiveness. This visually confirms the high overall rating of this sample, which was also evident earlier in the boxplot.

Samples A and D, on the other hand, score highly in terms of cheese flavour, meat aroma and colour characteristics, which correlates with earlier results, where these samples excelled in parameters related to the addition of cheese. Samples B and C have less complex and less consistent profiles, with clear weaknesses in terms of seasoning flavour, cheese aroma and cohesiveness, among others.

The radar chart, combining all characteristics in a single profile, allows for the assessment of the balance between individual attributes and complements the earlier presentation of differences in the form of a boxplot. The radar chart is one of the most transparent tools for presenting data from sensory profile analysis and is consistent with the assumptions of QDA [36,37].

## 4. Conclusions

This study provided a comparative instrumental and sensory characterisation of commercially available cheese-containing frankfurters from the Polish market. The results indicated associations between cheese content and certain quality attributes, including texture, colour, and flavour.

However, the relationship between cheese addition and texture was not linear. Products with similar cheese levels showed different hardness and springiness depending on cheese type and formulation matrix. For instance, high-cheese samples (A and D, 12%) displayed intermediate hardness, while the softest (C) and firmest (E) products contained 5% and 8% cheese, respectively.

This variability suggests that the final texture depends on the interplay among cheese composition, fat and moisture dynamics, and the overall meat matrix, rather than on cheese content alone. Cheese addition modifies the structure and sensory perception of frankfurters, but its influence must be considered in the context of formulation synergy.

Future work should include controlled formulations and consumer acceptance testing to verify these associations and better understand the technological role of cheese in processed meat systems.

## Figures and Tables

**Table 1:** Ingredients of cheese-added frankfurters as declared by the manufacturer.

Brand	Ingredients
A	Frankfurter 88% [pork ham meat 95%, starch, salt, spices, spice extracts, smoke flavouring, sugar, pork protein, triphosphates, monosodium glutamate, sodium ascorbate, sodium nitrite]. Processed cheddar cheese 12% [cheddar cheese 44%, water, milk, whey proteins, polyphosphates, sodium phosphates, salt, paprika extract, carotenes].
B	Pork 72%, water, hard cheese, salt, potato starch, dextrose, diphosphates, ascorbic acid, spices, spice extracts, sodium nitrite, beech wood smoke.
C	Pork 49%, chicken meat 19%, water, La Maar cheese 5% (rennet, bacterial cultures, salt, potassium nitrate), salt, potato starch, pork collagen protein, lactose and cheese powder, glucose, spices, spice extracts, sodium nitrite.
D	Frankfurter 88% [pork ham meat 95%, starch, salt, spices, spice extracts, smoke flavouring, sugar, pork protein, triphosphates, monosodium glutamate, sodium ascorbate, sodium nitrite]. Processed cheddar cheese 12% [cheddar cheese 44%, water, milk, whey proteins, polyphosphates, sodium phosphates, salt, paprika extract, carotenes].
E	Chicken meat 37%, pork meat 9%, beef meat 6%, water, ripened cheese 8% (pasteurised milk, salt, rennet, microbial cultures, calcium chloride, annatto), pork fat, chicken connective tissue, chicken fat, potato starch, salt, beef connective tissue, beef fat, triphosphates, sodium alginate, calcium sulphate, diphosphates, sodium ascorbate, monosodium glutamate, dextrose, ascorbic acid, sodium isoascorbate, glucose, maltodextrin, carminic acid, sodium nitrite.

**Table 2:** Mean Warner–Bratzler shear force and shear work values.

Brand	Shear Force [N]	Shear Work [N·s]
A	2.74 ^abc^ ± 0.35	8.87 ^a^ ± 1.04
B	2.91 ^b^ ± 0.38	9.81 ^a^ ± 1.17
C	1.82 ^d^ ± 0.35	5.72 ^b^ ± 0.32
D	2.63 ^abc^ ± 0.52	9.18 ^a^ ± 0.99
E	2.35 ^c^ ± 0.15	7.71 ^c^ ± 0.77

Mean values marked with different letters in the column differ significantly (*p* ≤ 0.05). The results are presented as mean ± standard deviation.

**Table 3:** Texture discriminant values in the TPA study.

Discriminant	A	B	C	D	E
Hardness 1 [N]	15.86 ^ab^ ± 3.84	21.08 ^a^ ± 6.67	11.47 ^b^ ± 4.13	16.04 ^ab^ ± 2.87	17.61 ^ab^ ± 2.24
Hardness 2 [N]	13.57 ^ab^ ± 4.2	16.34 ^a^ ± 7.79	10.34 ^b^ ± 4.07	12.39 ^ab^ ± 2.21	14.6 ^ab^ ± 1.32
Adhesiveness [N·s]	−0.379 ^a^ ± 0.211	−0.104 ^a^ ± 0.091	−0.141 ^a^ ± 0.192	−0.276 ^a^ ± 0.277	−0.183 ^a^ ± 0.067
Springiness	0.805 ^a^ ± 0.076	0.815 ^a^ ± 0.114	0.988 ^c^ ± 0.020	0.863 ^a^ ± 0.137	0.951 ^b^ ± 0.084
Cohesiveness	0.462 ^a^ ± 0.065	0.488 ^a^ ± 0.203	0.713 ^b^ ± 0.133	0.524 ^a^ ± 0.065	0.597 ^a^ ± 0.048
Gumminess	7.409 ^a^ ± 2.443	9.990 ^a^ ± 8.132	8.386 ^a^ ± 4.215	9.502 ^a^ ± 3.542	10.137 ^a^ ± 1.183
Chewiness	5.971 ^a^ ± 2.793	7.733 ^a^ ± 8.905	8.498 ^a^ ± 5.135	7.512 ^a^ ± 4.056	9.443 ^a^ ± 1.844
Resilience	0.205 ^a^ ± 0.048	0.240 ^a^ ± 0.163	0.366 ^b^ ± 0.086	0.242 ^a^ ± 0.036	0.297 ^c^ ± 0.037

Median values marked with different letters in rows differ significantly at (*p* ≤ 0.05). The Results are presented as median ± interquartile range.

**Table 4:** Colour of cheese-added frankfurters in CIE L*a*b* space.

Brand	L*	a*	b*
A	41.25 ^a^ ± 12.23	9.55 ^a^ ± 1.20	30.10 ^a^ ± 9.77
B	53.75 ^b^ ± 4.80	5.30 ^b^ ± 3.18	12.70 ^ab^ ± 0.72
C	57.40 ^b^ ± 3.70	4.40 ^bc^ ± 0.15	10.70 ^b^ ± 0.95
D	51.95 ^ab^ ± 6.70	8.35 ^ad^ ± 2.10	22.35 ^a^ ± 13.20
E	52.00 ^b^ ± 8.58	4.85 ^bd^ ± 0.25	10.15 ^b^ ± 0.85

Median values marked with different letters in the columns differ significantly at (*p* ≤ 0.05). The Results are presented as median ± interquartile range.

**Table 5:** Results of quantitative descriptive analysis of cheese-containing frankfurters.

Attribute	Descriptor	A	B	C	D	E
Colour	Colour uniformity	7.8 ^a^ ± 8.0	7.8 ^a^ ± 2.6	7.9 ^a^ ± 4.9	7.8 ^a^ ± 8.0	7.8 ^a^ ± 2.1
	Colour intensity	8.2 ^a^ ± 0.9	7.5 ^ab^ ± 1.4	4.7 ^b^ ± 2.0	8.2 ^a^ ± 0.4	6.3 ^b^ ± 1.3
Texture	Hardness	4.8 ^a^ ± 1.9	5.2 ^a^ ± 1.9	3.4 ^a^ ± 1.6	4.7 ^a^ ± 1.2	4.5 ^a^ ± 1.4
	Graininess	3.2 ^a^ ± 3.0	2.8 ^a^ ± 2.9	1.0 ^a^ ± 2.4	2.1 ^a^ ± 2.9	1.2 ^a^ ± 3.0
	Cohesiveness	6.3 ^a^ ± 3.4	6.7 ^a^ ± 3.3	5.2 ^a^ ± 1.8	6.0 ^a^ ± 3.1	5.9 ^a^ ± 3.2
	Juiciness	4.9 ^a^ ± 1.9	4.6 ^a^ ± 2.8	5.1 ^a^ ± 1.3	4.0 ^a^ ± 1.4	5.9 ^a^ ± 2.3
Flavour	Fat flavour	4.7 ^a^ ± 2.3	4.2 ^a^ ± 2.2	4.0 ^a^ ± 1.2	4.0 ^a^ ± 2.9	4.0 ^a^ ± 1.3
	Meat flavour	3.2 ^a^ ± 2.3	4.7 ^a^ ± 1.9	2.8 ^a^ ± 2.4	4.0 ^a^ ± 2.4	4.7 ^a^ ± 2.5
	Salty flavour	4.3 ^a^ ± 2.3	5.1 ^a^ ± 1.5	4.8 ^a^ ± 2.5	4.7 ^a^ ± 1.1	4.3 ^a^ ± 1.3
	Off-taste	0.3 ^a^ ± 1.4	0.2 ^a^ ± 1.0	0.5 ^a^ ± 2.3	0.2 ^a^ ± 1.2	0.2 ^a^ ± 1.1
	Seasoning flavour	3.0 ^a^ ± 3.5	4.8 ^a^ ± 1.4	4.1 ^a^ ± 2.8	4.1 ^a^ ± 2.5	5.8 ^a^ ± 3.5
	Smoky flavour	2.2 ^a^ ± 1.7	4.5 ^a^ ± 1.8	4.7 ^a^ ± 5.7	2.3 ^a^ ± 1.8	4.7 ^a^ ± 2.1
	Cheese flavour	6.9 ^a^ ± 1.5	2.0 ^b^ ± 4.9	2.2 ^ab^ ± 6.1	6.7 ^ab^ ± 1.4	2.6 ^ab^ ± 2.6
Aroma	Fat aroma	2.9 ^a^ ± 2.6	3.5 ^a^ ± 2.5	2.9 ^a^ ± 2.5	2.9 ^a^ ± 2.6	4.3 ^a^ ± 3.1
	Meat aroma	3.4 ^a^ ± 1.8	4.5 ^a^ ± 1.1	3.3 ^a^ ± 2.3	3.2 ^a^ ± 2.8	4.8 ^a^ ± 2.7
	Smoky aroma	4.0 ^a^ ± 1.5	4.3 ^a^ ± 0.9	4.7 ^a^ ± 0.7	3.7 ^a^ ± 1.4	4.5 ^a^ ± 1.0
	Seasoning aroma	2.8 ^a^ ± 2.7	4.9 ^a^ ± 2.9	3.3 ^a^ ± 3.0	2.0 ^a^ ± 2.5	4.8 ^a^ ± 3.6
	Cheese aroma	7.5 ^a^ ± 2.5	3.9 ^be^ ± 3.6	1.5 ^ce^ ± 1.3	6.2 ^abd^ ± 1.9	2.5 ^be^ ± 3.4
	Off-aroma	0.2 ^a^ ± 0.6	0.2 ^a^ ± 0.5	0.6 ^a^ ± 0.8	0.6 ^a^ ± 1.7	0.2 ^a^ ± 1.2
Overall evaluation		4.4 ^a^ ± 3.4	6.0 ^b^ ± 1.5	4.4 ^a^ ± 6.0	4.6 ^a^ ± 2.2	7.8 ^b^ ± 1.6

Median values marked with different letters in rows differ significantly (*p* ≤ 0.05). The Results are presented as median ± interquartile range.

## Data Availability

The original contributions presented in the study are included in the article; further inquiries can be directed to the corresponding author.

## Abbreviations

The following abbreviations are used in this manuscript:

WBSF	Warner–Bratzler Shear
TPA	Texture Profile Analysis
QDA	Quantitative Descriptive Analysis
ANOVA	Analysis of variance
